# Late-Onset Neutropenia Induced by Rituximab in Rheumatic Diseases: A Report of Two Cases of Severe Presentation and a Literature Review

**DOI:** 10.7759/cureus.80074

**Published:** 2025-03-05

**Authors:** Giulia Fernandes M Trevisani, Vanessa Furtado V Bento, Nilton Salles Rosa Neto

**Affiliations:** 1 Internal Medicine, Universidade Santo Amaro, São Paulo, BRA; 2 Center for Rare and Immune Disorders, Hospital Nove de Julho, São Paulo, BRA; 3 Rheumatology, Universidade Santo Amaro, São Paulo, BRA

**Keywords:** case report, immune-mediated disease, neutropenia, rheumatoid arthritis, rituximab, systemic lupus erythematosus, systemic vasculitis

## Abstract

Rituximab, a chimeric monoclonal antibody targeting the CD20 antigen on B cells, is widely used in oncological and immune-mediated diseases. However, late-onset neutropenia can occur, even in patients receiving concomitant immunosuppressants or chemotherapeutics, necessitating therapeutic adjustments. The development of neutropenia with rituximab monotherapy reinforces the relationship, but the exact pharmacological mechanism is still unknown. We report two cases of late-onset neutropenia after rituximab therapy: the first case is related to a woman with rheumatoid arthritis and anti-neutrophil cytoplasmic antibodies (ANCA)-associated vasculitis overlap and a history of alveolar hemorrhage; the second case concerns a woman with systemic lupus erythematosus and neurological manifestations. Both patients were hospitalized for febrile neutropenia, an unusual complication, and subsequently recovered after treatment with antibiotics and granulocyte colony-stimulating factor. Rituximab was discontinued. It is essential for rheumatologists to recognize and monitor for late-onset neutropenia during and after rituximab treatment, as early detection and intervention can prevent severe complications. The heterogeneity in clinical course observed in reported cases underscores the complexity of the condition and the impact on patient safety. The feasibility of resuming rituximab treatment after late-onset neutropenia requires careful evaluation.

## Introduction

Rituximab (RTX) is a chimeric monoclonal antibody that specifically interacts with the CD20 antigen present in B cells, from the pre-B stage to mature B lymphocytes. The mechanism of action of RTX is B-cell lysis through (I) antibody-dependent cell-mediated cytotoxicity, (II) antibody-dependent cellular phagocytosis, (III) complement direct cytotoxicity, and (IV) transmembrane signaling and apoptosis induction [1,2]. Since 1997, RTX has been approved for several conditions, either oncological, such as non-Hodgkin's lymphoma and chronic lymphocytic leukemia, or non-oncological, such as rheumatoid arthritis (RA), granulomatosis with polyangiitis (GPA), microscopic polyangiitis (MPA), and pemphigus vulgaris [2,3].

The understanding of B-cell involvement in other immune-mediated diseases has led to the off-label use of RTX in conditions such as autoimmune hemolytic anemia, systemic lupus erythematosus (SLE), and immune thrombocytopenic purpura [1-3]. Thus, RTX is one of the most prescribed medications for conditions without an approved indication in the package insert [4]. Despite being well tolerated by most patients, with the expansion of its use, an increasing number of adverse effects have been recognized.

Late-onset neutropenia (LON) is a condition where neutropenia develops weeks or months after treatment, most often with anti-CD20 drugs such as RTX or ocrelizumab. It is defined as neutropenia occurring at least four weeks after the last administration of the causal drug. It can occur in up to 30% of patients treated with RTX, more frequently in diseases such as SLE and lymphoma [3,5-8]. Neutropenia is defined by the National Cancer Institute Common Toxicity Criteria as an absolute neutrophil count <1.5 x 10^9^/L (neutropenia grades II to IV) [9]. The pathophysiology of neutropenia in this setting is uncertain and may vary according to the underlying disease and concomitant medications, but usually, there is no alternative explanation for the neutropenia [10]. A common complication is febrile neutropenia, which is defined as the presence of fever (temperature greater than 100.3°F or 37.9°C) plus an absolute neutrophil count of less than 0.5 x 10^9^/L [11,12]. The clinical significance of LON is substantial because it may impact treatment strategies [6].

There are many reports of LON resulting from RTX treatment for immune-mediated diseases managed by the rheumatologist. Although it can recuperate spontaneously and be unnoticed, LON can predispose to severe infections [10,13-20]. We report two cases of patients with LON after RTX therapy who were hospitalized because of febrile neutropenia, an uncommon complication. They both recovered uneventfully but required modifications to their therapeutic regimens.

## Case presentation

This case report was approved by the Universidade Santo Amaro Ethics Review Board, São Paulo, Brazil (approval number: 6.680.816). Both patients reviewed and signed the informed consent. The procedures adhered to the ethical standards of the responsible committee on human experimentation and the Helsinki Declaration of 1975, as revised in 1983. We followed the 2013 CARE (Consequences, Alternatives, Reality, and External Factors) checklist for case reports, which is available in the Appendices.

Case one

A 56-year-old woman was diagnosed with RA in 2008 and was treated with methotrexate, which was prescribed as MTX at a maximum dose of 25 mg/week. Treatment was temporarily discontinued in 2010 due to a colon cancer diagnosis, which required proctosigmoidectomy. Methotrexate was later reintroduced. She also had a prior history of breast cancer, in remission, status post left mastectomy in 1998. In 2014, she was diagnosed with interstitial lung disease, and a lung biopsy revealed poorly formed granulomas. This finding was interpreted at the time to be methotrexate-induced pneumonitis. The drug was discontinued, and the patient was referred for a second rheumatology opinion.

The patient remained on low-dose prednisone and was monitored for disease activity. At the first visit, the patient did not have active synovitis; laboratory results were positive for rheumatoid factor and anti-SSA antibody and negative for anti-citrullinated protein antibody. She complained of dry eyes and mouth, compatible with secondary Sjögren's disease.

At a follow-up visit, she presented with acute symptoms of fatigue, tiredness, and laboratory results revealing anemia. The patient was referred to the emergency department, and a chest computed tomography (CT) scan revealed bilateral ground-glass opacities, predominantly on the right, a nodule in the left apex, fibrotic septa with bronchiectasis in the left upper lobe, centrilobular opacities, and ground-glass opacities in the middle lobe. Bronchoalveolar lavage confirmed alveolar hemorrhage; the results were negative for bacteria, fungi, and mycobacteria, and lung biopsy revealed organizing pneumonia and no evidence of granuloma or necrosis. The rheumatoid factor was 16 U/mL (normal range: <14), and antinuclear antibody patterns were nuclear fine-speckled (AC-4) 1/160 and cytoplasmic reticular (AC-21) 1/320. The patient also tested positive for p-ANCA 1/40, while the anti-glomerular basement membrane was negative. Given the diagnosis of RA overlap with ANCA-associated vasculitis (AAV), alveolar hemorrhage, and a previous history of cancer and elevated risk of cancer recurrence, treatment was initiated with pulse therapy with methylprednisolone 1 g/day for three days, followed by two 1 g infusions of RTX 14 days apart (November and December 2014). RTX was then administered in May and June 2015 with significant improvement in her condition and steroid tapering.

In July 2015, she presented to the rheumatology clinic and reported having myalgia, fever, conjunctival hyperemia, and pain, edema, and erythema of the right foot secondary to a dermatophyte infection. Importantly, neutropenia (27 neutrophils/µL) (normal range: 1,800-7,800) was identified. She was admitted on the same day for further evaluation of febrile neutropenia with right foot cellulitis. Antibiotic therapy with ceftriaxone and oxacillin, together with oral fluconazole and topical isoconazole, was initiated, and she received subcutaneous filgrastim 300 mg/day. Blood cultures were negative. Serologic tests for toxoplasmosis, hepatitis B, hepatitis C, syphilis, and HIV were negative. Epstein-Barr virus, cytomegalovirus, dengue fever, and hepatitis A were IgG-positive and IgM-negative. Anti-dsDNA was negative, complement levels were within the normal range (C3: 127 mg/dL and C4: 30 mg/dL), and ferritin was 150 ng/mL (normal range: 30-300). Neutrophil levels increased after three days, and she completed a seven-day course of antibiotics. Table 1 describes the complete blood count results for patient one during hospital admission.

**Table 1:** Laboratory parameters of patient one during hospital admission (2015)

Parameter	Reference Range	July 2015				
		6	7	8	9	12
Hemoglobin (g/dL)	12–15	12.5	12.2	12.2	11.8	11.4
Leukocytes (/µL)	4,000–10,000	2,700	2,800	7,000	12,000	6,600
Neutrophils (/µL)	1,800–7,800	27	168	1,610	6,120	2,772
Immature neutrophils (/µL)	0–3%	-	1	4.5	2.4	11
Lymphocytes (/µL)	1,000–3,000	2,160	2,100	910	2,160	2,376
Platelets (/µL)	150,000–450,000	205,000	213,000	206,000	246,000	198,000

**Figure 1:**
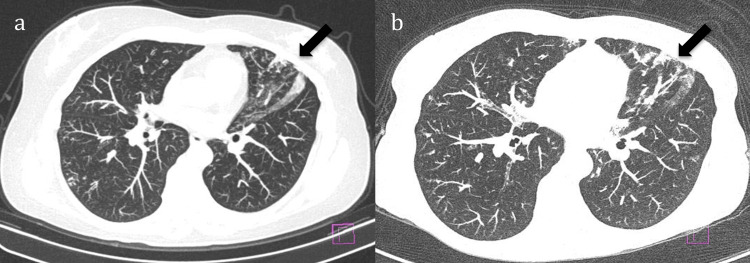
Lung interstitial findings (arrows) from chest CT scans obtained in 2016 (a) and 2024 (b)

She was discharged following a complete recovery. The interval between the last dose of RTX and the onset of neutropenia was four weeks, which aligns with the diagnosis of LON. She received a total of four infusions (cumulative dose 4 g). Treatment with RTX was discontinued, and she remained off immunosuppressive therapy until 2024 without evidence of disease recurrence, neither articular nor pulmonary. There was no recurrence of neutropenia. Figure 1 presents stable lung interstitial findings from sequential chest CT scans.

Case two

A 45-year-old woman was diagnosed in 2010 with SLE. She manifested cutaneous, articular, and renal involvement, as well as peripheral neuropathy. She had been treated with azathioprine, hydroxychloroquine, and steroids. Anti-dsDNA was persistently positive. She also had a history of deep vein thrombosis in the left lower limb, pulmonary embolism, and positive anticardiolipin antibodies. Anticoagulation was started, but the target international normalized ratio of 2.0 to 3.0 was difficult to achieve due to interaction with azathioprine. Because of progressive walking impairment, RTX and mycophenolate mofetil (MMF) 3 g/day were initiated in 2013. She received two 1 g infusions 14 days apart at every cycle. Improvement in walking and balance was noted, and she received subsequent cycles of RTX.

**Figure 2:**
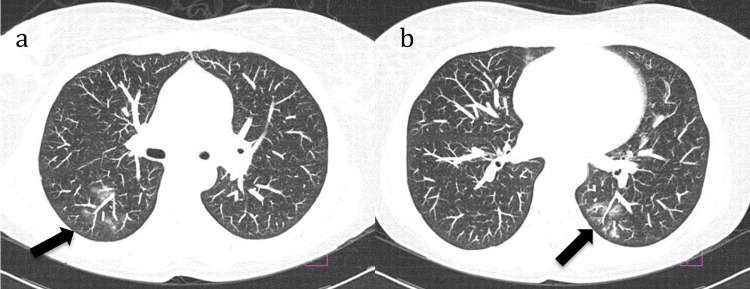
Ground-glass opacities (arrows) from chest CT (2016) (a, b)

In July 2016, the patient presented to the emergency department with a three-day history of odynophagia, accompanied by dry cough, clear rhinorrhea, and fever. On physical examination, only erythema of the oropharynx was noted, without plaques or palatal petechiae, but chronic tongue lesions and painful bilateral submandibular lymphadenopathy, worse on the left, were noted. A complete blood count was ordered and revealed leukopenia (700 leukocytes/µL) (normal range: 4,000-10,000), neutropenia (unmeasurable), and elevated C-reactive protein (CRP), leading to a diagnosis of febrile neutropenia. The investigation included negative serologic results for erythrovirus B19, cytomegalovirus, and Epstein-Barr virus and a negative respiratory viral panel. Chest CT showed ground-glass opacities suggesting infection (Figure 2). A possible SLE flare was less likely because of stable anti-dsDNA titer 1/40 and normal complement levels. She received her last RTX cycle in January 2016. The interval between the previous dose of RTX and the onset of neutropenia was 24 weeks. She received a total of eight infusions (cumulative dose of 8 g).

Treatment with cefepime, as per the local protocol associated with subcutaneous filgrastim 300 mg/day, was started, and MMF was stopped. A daily complete blood count was ordered for monitoring. Within two days, the patient showed considerable improvement, with an increase in the leukocyte count and a decrease in CRP. She was discharged on hydroxychloroquine 400 mg/daily and MMF 500 mg/daily.

Despite this serious event, the next planned cycle of RTX (two 1 g infusions 14 days apart) was uneventfully administered in November 2016. However, in December 2016, there was a recurrence of neutropenia, this time accompanied by chronic diarrhea. The interval between the last dose of RTX and the onset of neutropenia was four weeks, which aligns with the diagnosis of LON. The anti-dsDNA was positive (1/20), and there was a decrease in complement levels (C3: 49 mg/dL and C4: 11 mg/dL). Microbiologic testing was negative for parasites, rotavirus, *Clostridioides difficile*, and *Giardia lamblia*. Celiac disease evaluation was negative, including anti-gliadin IgA and IgG and anti-endomysial IgA. Upper endoscopy and colonoscopy were normal, and there was no evidence of intestinal cytomegalovirus infection, atrophic mucosa, or lymphocyte infiltration. Immunoglobulin levels (mg/dL) were within the normal range (IgA: 282; IgM: 97; IgG: 1180). The possible link between neutropenia and the prior use of RTX was then considered despite evidence of SLE reactivation. The prednisone dose was increased, and she received subcutaneous filgrastim 300 mg/day. She was discharged following leukocyte recovery. Tables 2, 3 describe the complete blood count results for patient two during hospital admissions in July and December 2016, respectively.

**Table 2:** Laboratory parameters of patient two during hospital admission in July 2016

Parameter	Reference Range	July 2016				
		5	6	7	8	10
Hemoglobin (g/dL)	12–15	13	12.2	12	12.6	11.7
Leukocytes (/µL)	4,000–10,000	700	500	900	2,700	5,200
Neutrophils (/µL)	1,800–7,800	-	-	-	1,323	3,432
Immature neutrophils (/µL)	0–3%	-	-	-	5	4
Lymphocytes (/µL)	1,000–3,000	-	-	-	1,026	1,040
Platelets (/µL)	150,000–450,000	237,000	203,000	290,000	225,000	204,000

**Table 3:** Laboratory parameters of patient two during hospital admission in December 2016

Parameter	Reference Range	December 2016				
		13	14	17	18	19
Hemoglobin (g/dL)	12–15	13.8	13.3	13.1	12.9	12.8
Leukocytes (/µL)	4,000–10,000	1,800	1,400	1,400	2,100	2,500
Neutrophils (/µL)	1,800–7,800	1,080	672	336	1,176	1,125
Immature neutrophils (/µL)	0–3%	2	2	3	13	5
Lymphocytes (/µL)	1,000–3,000	468	448	770	399	750
Platelets (/µL)	150,000–450,000	266,000	234,000	251,000	237,000	248,000

In March 2017, RTX was switched to intravenous monthly belimumab at a dosage of 10 mg/kg due to the SLE flare, and she received this treatment periodically until 2020. Neutropenia did not recur.

## Discussion

We describe the occurrence of LON following RTX infusions in two patients with rheumatological immune-mediated diseases: the first with overlap syndrome, RA and AAV, and the second with SLE. Both developed febrile neutropenia, recovered, and required treatment modification due to adverse events.

The exact mechanism behind the development of LON after RTX treatment is still uncertain, but two main theories are considered: immune-mediated reactions and hematopoietic disturbances following RTX therapy. The concept of lymphocyte imbalance leading to LON was proposed based on the presence of large granular lymphocytes in the peripheral blood and bone marrow of affected patients. Variations in stromal-derived factor-1 and the cytokine BAFF (B-cell-activating factor) have also been suggested as potential contributors to the pathogenesis of LON [10,15,18]. A study linked the FCγRIIIa (FCGR3A)158 V genetic variant to higher LON rates [21]. Recently, Tashiro et al. assessed the outcomes of kidney transplants and the genotype for the FCGR3A 158 variant. A multivariate analysis corroborated the V-allele as an independent risk factor for LON. Of note, no significant differences in the incidence rates of post-transplant infection and rejection between the FF and FV + VV genotypes were seen [22]. Additional hypotheses comprise CD20-trogocytosis and antibody-mediated cytotoxicity, usually related to early-onset neutropenia, which could partly explain some of the protracted cases; the removal of neutropoietic growth factors (granulocyte-macrophage colony-stimulating factor (GM-CSF) and granulocyte colony-stimulating factor (G-CSF)) due to B-cell depletion and bone marrow rerouting associated with B-cell recovery, where bone marrow production is biased toward B cells in detriment of neutrophils [23].

The incidence and clinical patterns of post-RTX LON vary across different diseases, both oncological and immune-mediated. In patients with SLE treated with RTX, the frequency of LON was 29.9%, with most cases being self-limited and asymptomatic. In contrast, the incidence of LON in AAV is estimated at 11%, in RA at 1.3-4.6%, and in lymphoma treatment, it ranges from 5.6% to 27.3%. It can be said that the risk of LON is approximately three times higher in SLE patients compared to those with AAV. However, the incidence data may be underestimated, as patients with transitory, asymptomatic neutropenia may not be included [6,7,19].

Several reports have tried to find risk factors for LON, but due to patient heterogeneity, no consensus has been reached. Nevertheless, receiving more than four doses of RTX and prior chemotherapy are considered potential risk factors. In addition to the variability in LON incidence, it is seen that the clinical course in patients receiving concomitant immunosuppressive treatment appears to differ from that in lymphoma patients undergoing standard chemotherapy. On the other hand, the development of LON raises important questions about its clinical relevance, such as whether there is an increased risk of infections and whether it is safe to resume RTX treatment in affected patients [3,5,6,13,14]. One of our patients had previous cancer treatments and received conventional disease-modifying antirheumatic drugs for RA. We opted not to use cyclophosphamide, and she was treated with a combination of RTX and steroids. The second patient had failed multiple therapies for SLE before starting a combination of MMF and RTX. Because of the infectious risk, both patients stopped RTX.

Most patients with LON remain asymptomatic and are often discovered incidentally during routine laboratory tests. In contrast, infections were observed in all symptomatic patients, with LON identified from a complete blood count due to the onset of unexpected symptoms. Severe cases of LON can progress to life-threatening complications such as sepsis, emphasizing the need for close neutrophil count monitoring, infection surveillance, and early initiation of broad-spectrum antibiotics in febrile patients. There are no clear guidelines about the need for GM-CSF therapy in patients with LON. It is generally believed that in most cases of grade III RTX-associated neutropenia, neutrophils return to their normal value, and patients recover quickly without specific therapy or GM-CSF. On the other hand, some patients with infectious complications may require GM-CSF for treatment, as was the case in both our patients [3,16,19]. A case series of patients with non-malignant hematological autoimmune disorders treated with RTX found an 18% rate of neutropenia in one year, mostly mild, and that the treatment combination with other immunosuppressants increased the chance of more severe neutropenia and longer time to recovery. They reported a low number of hospitalizations, infections, and use of GM-CSF [24].

Of note, the duration of empiric therapy for febrile neutropenia varies between guidelines. It is usually recommended to discontinue empiric antimicrobial treatment when the absolute neutrophil count is > 0.5 x 10^9^/L and increasing, the patient has been afebrile and asymptomatic for at least 48 hours, and blood cultures are negative [12]. Our patients were treated with intravenous antibiotics for at least seven days and with GM-CSF until their absolute neutrophil count recovered.

The occurrence of severe adverse events, such as LON with RTX, underscores the need for heightened clinical vigilance in patients receiving immunomodulatory or targeted therapies. Recognizing severe complications of biologic therapies is essential for patient safety. This study reinforces the need for ongoing research and post-marketing surveillance to enhance drug safety profiles while highlighting the importance of comprehensive patient monitoring, timely diagnosis, and patient education to prevent fatal outcomes. Our experience is to obtain a complete blood count from the treated patients one month after the last dose of the cycle and every three to four months until the next programmed dose.

Our work has limitations. We did not obtain bone marrow aspiration or biopsy, so we cannot explore the possible underlying hematopoietic mechanisms involved in these two cases. However, we had a long follow-up period after these events for both patients, and neutrophil counts were not impacted by new therapies or infections, reinforcing the likelihood of RTX as a causal agent of neutropenia.

## Conclusions

In conclusion, LON following RTX therapy is a significant complication in patients with immune-mediated rheumatological diseases. Recognition and proactive management of this potential side effect are crucial for rheumatologists. Routine neutrophil count monitoring, patient education on infection symptoms, and individualized risk assessment can mitigate the risk of severe adverse events.

## Figures and Tables

**Figure 3:**
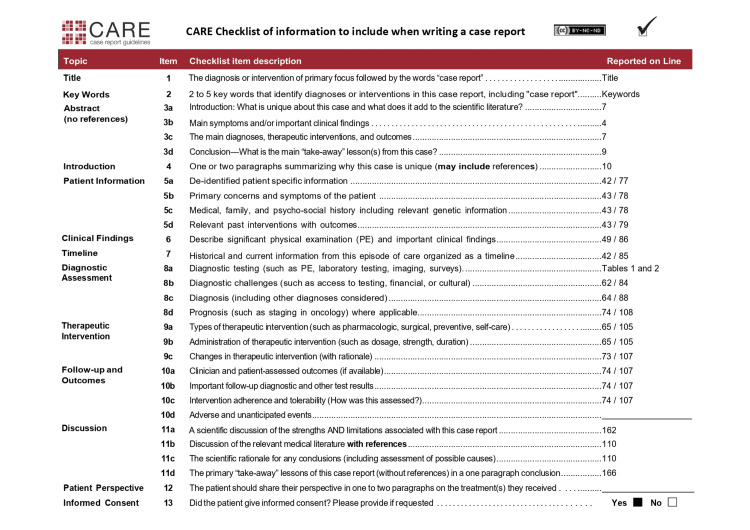
CARE checklist CARE: Consequences, Alternatives, Reality, and External Factors Source: https://www.care-statement.org/checklist
